# Clinical Mycology Today: Emerging Challenges and Opportunities

**DOI:** 10.1093/ofid/ofae363

**Published:** 2024-06-27

**Authors:** Jessica Little, Adriana M Rauseo, Julio C Zuniga-Moya, Andrej Spec, Peter Pappas, John Perfect, Todd McCarthy, Ilan S Schwartz

**Affiliations:** Division of Infectious Diseases, Brigham and Women's Hospital, Harvard Medical School, Boston, Massachusetts, USA; Stem Cell Transplant and Cellular Therapy, Dana-Farber Cancer Institute, Harvard Medical School, Boston, Massachusetts, USA; Division of Infectious Diseases, Department of Medicine, Washington University School of Medicine, St. Louis, Missouri, USA; Division of Infectious Diseases, Department of Medicine, Washington University School of Medicine, St. Louis, Missouri, USA; Division of Infectious Diseases, Department of Medicine, Washington University School of Medicine, St. Louis, Missouri, USA; Division of Infectious Diseases, Department of Medicine, The University of Alabama at Birmingham, Birmingham, Alabama, USA; Division of Infectious Diseases, Department of Medicine, Duke University School of Medicine, Durham, North Carolina, USA; Division of Infectious Diseases, Department of Medicine, The University of Alabama at Birmingham, Birmingham, Alabama, USA; Division of Infectious Diseases, Department of Medicine, Duke University School of Medicine, Durham, North Carolina, USA

**Keywords:** AMR, antifungals, clinical trials, diagnostics, fungal infection, mycoses, mycosis

## Abstract

The Mycoses Study Group Education and Research Consortium is a collective of clinicians, researchers, and educators with the common goal to advance awareness, diagnosis, and management of invasive fungal diseases. Clinical Mycology Today, the Mycoses Study Group Education and Research Consortium's biennial meeting, is dedicated to discussing the most pressing contemporary issues facing the field of clinical mycology, promoting clinical, translational, and basic science collaborations, and mentoring the next generation of clinical mycologists. Here, we review the current opportunities and challenges facing the field of mycology that arose from discussions at the 2022 meeting, with emphasis on novel host risk factors, emerging resistant fungal pathogens, the evolving antifungal pipeline, and critical issues affecting the advancement of mycology research.

## Teaser

Clinical mycology is at a pivotal inflection point, with expanding patient populations at risk of fungal infections, emerging drug-resistant pathogens, and promising new antifungals on the horizon.

Invasive fungal diseases (IFDs) are associated with significant morbidity and mortality and pose a growing threat globally [1]. The Mycoses Study Group Education and Research Consortium (MSGERC) is a collective of clinicians, researchers, and educators dedicated to advancing awareness, diagnosis, and management of IFDs. Clinical Mycology Today 2022, MSGERC's biennial meeting, was held September 7–9, 2022, in Albuquerque, New Mexico, and brought together stakeholders to review challenges and outline a vision for the field. All 40 presentations given at the meeting were recorded and are freely and openly available on the MSGERC's public YouTube channel (https://msgerc.org/2022-biennial-meeting/). The report here highlights salient themes to emerge from the meeting and attempts to expand on these discussions to highlight challenges and opportunities in clinical mycology for the next decade.

It is a dynamic time for the field of clinical mycology. Advances in medical therapies continue to lead to increased numbers of patients at risk of IFD. These include patients receiving solid organ and stem cell transplantation, chimeric antigen receptor (CAR) T-cell therapy, monoclonal antibodies, and small molecule targeted therapies. At the same time, virulent, highly transmissible, and/or resistant fungal pathogens have spread globally, challenging public health and infection prevention and control efforts. After years of stagnation in the development of antifungals, driven by challenges in trial execution and design as well as the lack of financial incentives to bring novel antimicrobials to market, promising new treatments are now on the horizon, including several new classes of antifungal therapies with novel mechanisms of action, as well as refinements in drugs from existing classes with improved tolerability, pharmacokinetics, and ease of administration. Despite this, there are major challenges anticipated, including anticipated shortfalls related to the number of clinicians and scientists with expertise and interest in fungal disease, limited research funding opportunities, and regulatory challenges related to clinical trial design execution.

## HOST FACTORS: NOVEL IMMUNOTHERAPIES AND ADVANCES IN IMMUNOGENETICS

**Figure 1.**
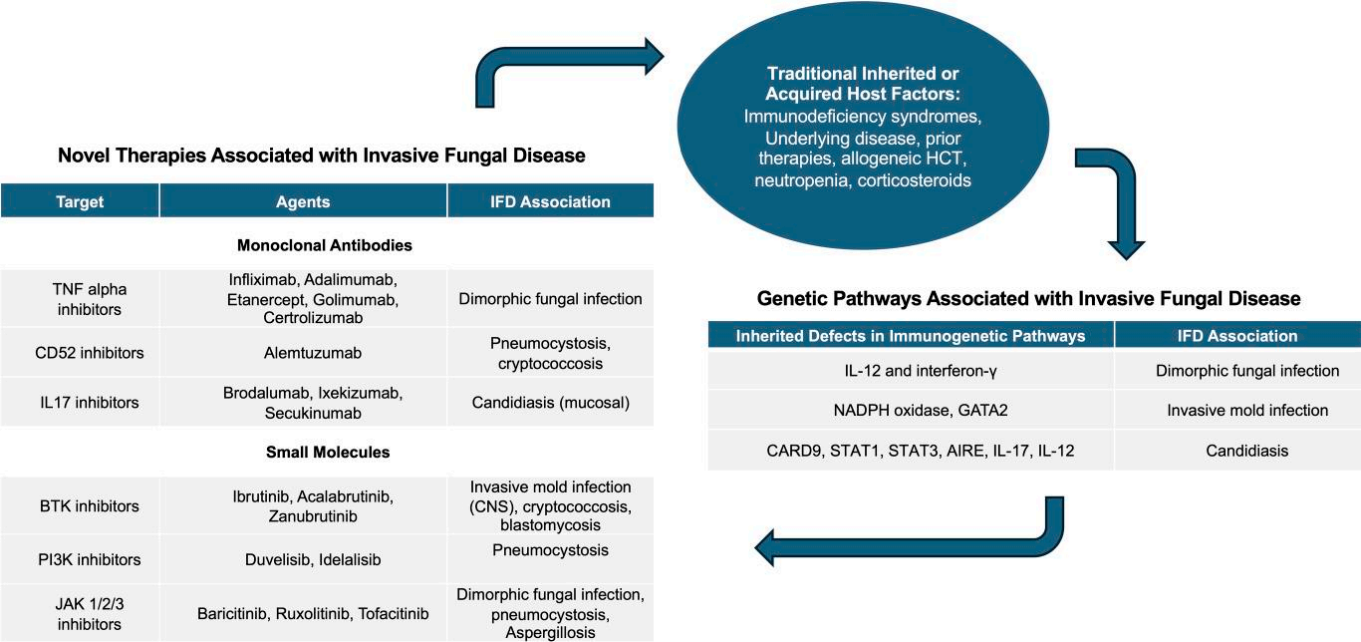
Interplay of risks of fungal disease from novel therapies, traditional inherited or acquired host factors, and genetic risk factors.

Host risk factors play a major role both in the development of fungal infections as well as clinical outcomes [2–8]. Patients with a weakened immune system are at increased risk for IFDs with specific predisposing host factors that include prolonged neutropenia, receipt of an allogeneic hematopoietic cell transplant (HCT), use of high-dose corticosteroids, administration of T-cell suppressive therapies, advanced HIV disease, and inherited immunodeficiencies [7–12]. However, over the past 2 decades, the epidemiology of IFDs has shifted, and newly acquired and inherited host risk factors have increasingly been recognized, as shown in Figure 1 [2, 13–18]. In particular, rapid growth of biologics and small molecule agents, as well as cellular therapies has occurred since the 1990s [19, 20]. These therapies target specific immunologic pathways or immune cell subsets as opposed to the broader immunosuppressive activity of corticosteroids or cytotoxic chemotherapy [21–23]. Despite this targeted approach, infections remain an important complication, and risk for individual pathogens can be difficult to predict based on mechanism. The speed with which these novel therapies are being developed and the lack of uniform attention to opportunistic infections in clinical trials has led to delayed identification of therapies that pose a risk for IFDs [24]. In addition to the development of novel agents, older biologic therapies with well-known risk profiles are being applied in new settings such as combination monoclonal antibody and small molecule therapy for chronic lymphocytic leukemia, maintenance immunotherapy following allogeneic HCT or CAR T-cell therapy, and use of Janus kinase inhibitors or tumor necrosis factor-α (TNF-α) inhibitors for treatment of graft-versus-host disease [25–32]. Longer durations of therapy are also being used, but the cumulative risk of infection over time has not been fully assessed in clinical trials. The risk of IFDs may be related to the patient's net state of immunosuppression more than to any individual agent and thus must continue to be reassessed with these new drug applications. Improved understanding of individual risk factors and the continued development of biomarkers and novel tools potentially harnessing the power of artificial intelligence to assess risk for IFDs in this population will be vital to prevent morbidity and mortality.

### Monoclonal Antibodies and Small Molecule Therapies

Monoclonal antibodies (Mabs) target cell surface receptors or soluble cytokines and are among the first immunotherapies approved in the United States [20]. Since the first MAbs approval in 1986, more than 100 products have surfaced across a multitude of fields [33, 34]. PD1/PDL1 immune checkpoint inhibitors, CD20 inhibitors, and TNF-α inhibitors constitute the most common targets. When assessing attributable risk for IFDs, it can be difficult to determine the impact of any individual therapy on a background of underlying disease, prior treatment regimens, and immunologic effects of combination therapy. In addition some agents, such as immune checkpoint inhibitors may be associated with acute toxicities that are in turn treated with additional immunosuppressive therapies such as corticosteroids, potentially increasing the risk of IFDs in those patients. Nonetheless, risk of IFDs appears to be low for these common Mabs [20, 21, 35, 36].

TNF-α inhibitors represent a notable exception with an increased risk for opportunistic infections reported in large studies including a predisposition for endemic fungal infections such as histoplasmosis and coccidioidomycosis, leading to a black box warning for these products [37–44]. Other less commonly used Mabs with clearly established risks for IFDs include CD52-targeted agents (alemtuzumab) and interleukin-17 (IL-17)–targeted agents (brodalumab, ixekizumab, and secukinumab) [21, 45]. Serious opportunistic infections including IFDs related to profound B- and T-cell depletion have been reported with alemtuzumab and routine *Pneumocystis jirovecii* pneumonia prophylaxis is recommended during use [46]. IL-17–targeted agents are primarily used for the treatment of autoimmune and inflammatory diseases and have been associated with an increased risk of candidiasis, which is predicted given the association of chronic mucocutaneous candidiasis with mutations affecting the IL-17 pathway [47–49]. Notably, invasive candidiasis appears to be uncommon and with these more mild mucosal infections, drug discontinuation is often unnecessary [49]. Finally, emerging evidence suggests that there may be an increased risk for IFDs in patients receiving belatacept (CTLA-4 monoclonal antibody) in patients following solid organ transplantation, though confirmatory studies are needed.

Small molecules represent another class of rapidly expanding targeted immunotherapy [50]. These agents typically act as intracellular secondary messengers and thus have a propensity for increased off-target effects including impaired pathogen response [20]. Several small molecule agents have been associated with an increased risk for IFDs. Bruton's tyrosine kinase inhibitors are the most well-known example, where multiple postmarketing studies emerged suggesting an increased risk for fungal infections, in particular with increased rates of invasive mold infections involving the central nervous system [19, 24, 51–60]. The risk appears to be increased for patients with chronic lymphocytic leukemia following prior antineoplastic therapy or receiving combination therapy with corticosteroids, demonstrating the impacts of cumulative immunosuppression [60, 61].

Phosphoinositide 3-kinase (PI3K) inhibitors are a second class of small molecule agents that have been associated with an increased risk for IFDs [22, 62]. These agents are primarily used for the treatment of hematologic malignancy, and multiple phase III studies have been halted because of high rates of death and serious adverse events that were primarily attributed to opportunistic infections including *Pneumocystis jirovecii* pneumonia [63–65]. Given more recent trials indicating worse overall survival for patients receiving PI3K inhibitors as well as significant toxicity, increased scrutiny is being placed on this drug class with several withdrawals from the market [66]. For patients receiving PI3K inhibitors, routine *Pneumocystis* prophylaxis is recommended and close surveillance for other opportunistic infections should be undertaken.

### Cellular Therapies

Cellular therapies such as CD19-directed CAR T-cell therapy for the treatment of acute lymphoblastic leukemia and large B-cell lymphoma, as well as B-cell maturation antigen-directed CAR T-cell therapy for treatment of multiple myeloma have thus far demonstrated a low risk of IFDs in multiple studies [67–69]. Cases have been reported and are often in the setting of advanced disease with extensive prior therapies including HCT or severe toxicities requiring treatment with high-dose corticosteroids [67, 70, 71]. Further studies are needed to characterize specific risk factors for IFDs in this population and to delineate the optimal approach to antifungal prophylaxis and stewardship in this population. Furthermore, as we begin to see increasing numbers of patients who are treated with cellular therapies earlier in the course of disease, or for nononcologic diseases, the risks of IFDs may be even lower and it will be important to continue to assess these risks and modify prophylactic strategies as indicated [72, 73].

### Genetic Risk for Invasive Fungal Disease

Advances in the molecular and genetic tools for assessment of acquired immunodeficiency have resulted in enormous gains in understanding of host genetic risk factors for IFDs over recent years. Genetic mutations in CARD9, STAT1, STAT3, and AIRE, as well as IL-17 and IL-12 receptors have been associated with chronic mucocutaneous candidiasis and invasive candidiasis as well as other fungal infections [18, 48, 74–76]. Invasive mold infections such as aspergillosis have been associated with defects in phagocyte effector function due to nicotinamide adenine dinucleotide phosphate oxidase mutations, as well as GATA2 mutations [18, 77, 78]. Finally, disruption of normal IL-12 and interferon-γ signaling appears to predispose patients to serious endemic or dimorphic fungal infections [17, 76, 79]. These insights have aided in better understanding the impacts of targeted therapies that act on similar pathways. Advances in the field of immunogenetics over the coming years may help to define genetic or immunologic markers that indicate an increased risk for IFDs in patients without diagnosed immunodeficiency syndromes [76, 80].

### Fungal Infections Complicating Respiratory Viral Infections

Even before the arrival of COVID-19, influenza-associated fungal infections were increasingly reported, albeit with significant heterogeneity in the prevalence across geographic regions. For instance, in a seminal retrospective study, the Dutch-Belgian Mycoses Study Group reported that aspergillosis complicated nearly 1 in 5 intensive care unit admissions for severe influenza [81]. COVID-19 has similarly introduced a large population of patients with localized and systemic immunological derangements that increase the likelihood of invasive fungal infection, even in persons without classic risk factors [82, 83]. Pulmonary aspergillosis is a serious complication that can compound morbidity and mortality of critically ill patients with COVID-19 and has been reported globally [83, 84]. In addition, in some geographic regions—most notably India—COVID-19 emerged as an important risk for rhino-orbital-cerebral and to a lesser extent pulmonary mucormycosis [85]. The public health burden from mucormycosis was substantial, with tens of thousands of persons in India affected during the delta wave, before the widespread availability of vaccines [85]. The most important risk factors for COVID-19-associated mucormycosis are poor glycemic control (often in persons with undiagnosed diabetes) worsened by receipt of corticosteroids [86].

### Iatrogenic Outbreaks of Fungal Disease

Some fungal infections are primarily acquired from the hospital environment, with prototypical examples being colonization and disease with *Candida auris*, or pulmonary or cutaneous mold infections in intensive care units or hematology wards when engineering controls are absent or compromised. In recent years, health care-associated outbreaks have emerged involving contaminated injectable medication. A decade ago, intrathecal injection of methylprednisolone from contaminated vials led to hundreds of cases of fungal meningitis caused by *Exserohilum rostratum* [87]. Far from being an isolated occurrence, there have been 2 recent outbreaks of iatrogenic fungal meningitis caused by *Fusarium sonali* linked to contaminated anaesthetics in Mexico [88, 89]. Prevention and containment of future national and international outbreaks will require ongoing cooperation between regulators, epidemiologists, laboratorians, and clinicians globally.

### Mitigating Emerging Host Risks

The landscape of host risk factors for IFD is rapidly transforming. As outlined here, it can be exceedingly difficult to characterize the attributable risk of any single therapeutic agent when administered on a background of advanced underlying disease and heavy prior immunosuppression. Nevertheless, it remains critical that there is ongoing focus on evaluating novel therapies with improved surveillance for IFDs in clinical trials, as well as continued evaluation in a “real-world” setting following approval. New applications of old drugs should not be considered to have the same risk profile as previously shown for other indications, and the cumulative immunosuppressive effects of long-term therapies should be considered.

## PATHOGENS: THE THREAT OF ANTIFUNGAL RESISTANCE

The landscape of fungal pathogens causing clinically important infections is diverse and dynamic. The most common and deadly pathogens have been collated and ranked by the World Health Organization in their first Fungal Priority Pathogen List, published soon after the meeting [1]. In addition to these familiar foes that continue to cause disease in hospital and community settings, pathogens with new epidemiological or geographical associations such as COVID-associated fungal disease have arisen in recent years [82]. In addition, fungal resistance patterns continue to shift with the emergence of drug-resistant fungal pathogens across multiple species and disease types [90]. This ongoing evolution of fungal pathogens has important implications for individual clinical management and treatment as well as global public health efforts.

### Drug-resistant Dermatophytes

Although not life-threatening, dermatophyte infections affect as many as 1 in 5 people around the world and can contribute to substantial morbidity through disfiguring and often painful or pruritic skin lesions [91]. In this context, drug-resistant dermatophytosis is an emerging global health threat [92]. Among *Trichophyton* species, the predominant agents of dermatophytosis in North America, *Trichophyton indotineae* (previously *T mentagrophytes* genotype VIII) has recently emerged as a novel strain with global spread, increasingly severe inflammatory lesions, and resistance to terbinafine and azole antifungals such as itraconazole and fluconazole, traditionally considered first-line treatments [93]. *Trichophyton rubrum* is the most common cause of dermatophytosis and has also had increasing treatment-resistance documented globally and in the United States [92]. Among dermatophytes referred to a U.S. reference laboratory in 2021–2022, Cañete-Gibas et al. reported that 18.6% were resistant to terbinafine, including isolates of *T rubrum* and *T indotineae* [93]. Furthermore, the incidence of resistant isolates may be underestimated given the challenges in obtaining antifungal susceptibilities with many cases not obtaining a confirmed microbiologic diagnosis [94]. These shifts in both the severity and susceptibility profiles of dermatophyte infections can significantly impact morbidity and have led to global outbreaks that are difficult to treat or control. Further studies are needed to better characterize the evolving epidemiology, transmission, host interactions, and outcomes of these challenging infections.

### Drug-resistant Aspergillosis

In contrast to dermatophytosis, invasive mold infections are less common but carry high mortality rates, occurring predominantly among immunocompromised persons. The most common cause of invasive mold infections is *Aspergillus fumigatus*, and among this species, azole resistance has increased in recent years. In some parts of the world, azole resistance in *A fumigatus* is clearly linked to use of azole fungicides in horticulture and agriculture but can also be impacted by patient exposure to antifungals via broadly increased use of antifungal prophylaxis in immunosuppressed populations [90, 95]. In the Netherlands, for example, as many as 15% of *A fumigatus* clinical isolates in some years are azole-resistant [96]. Rare cases of azole-resistant *A fumigatus* with telltale genetic mutations that bely this in-field mechanism of resistance acquisition have been reported in the United States, but clinical and environmental surveys have not identified rates of resistance that approach what is observed in the Netherlands [97]. Nonetheless, azole-resistance *A fumigatus* remains a real threat, with the potential for increased toxicity related to non-azole antifungal therapy and increased mortality reported in multiple studies [98].

In addition to *A fumigatus*, other species with reduced susceptibility to antifungals may also be implicated in disease. For example, *A terreus* is frequently resistant to amphotericin B [99]. In addition, cryptic species of *Aspergillus* with reduced susceptibility to antifungals can be overlooked by phenotypic identification methods.

### Candida auris

*Candida auris* is a multidrug resistant yeast that is prone to environmental colonization leading to outbreaks in the healthcare setting that are difficult to contain [100, 101]. *C auris* emerged independently in multiple regions in 2009 and has continued to spread around the world and across North America [102]. The COVID-19 pandemic “was like throwing a bit of lighter fluid on the fire” noted Centers for Disease Control and Prevention Mycotic Diseases Branch Chief, Dr. Tom Chiller, at the meeting. In the United States, clinical cases of *C auris* increased dramatically, with year-over-year increase of 95% between 2020 and 2021 [100]. Major infection prevention and control challenges caused by *C auris* remain, including difficulty of disinfecting contaminated health care environments, and inability to effectively decolonize patients.

## TREATMENT: NEW HOPE ON THE HORIZON

After years of stagnation, the antifungal development pipeline is flourishing [103, 104]. There are new antifungal agents with meaningful advantages in pharmacokinetic properties, safety profile, and/or route of administration compared to extant options from existing antifungal classes, as well as first-in-class antifungals with novel mechanisms of action that are in advanced stages of development (Table 1) [105]. In addition, investigation of the potential benefits of immunotherapy or immune augmentation as an adjunctive approach to standard antifungal therapy in patients with IFDs is ongoing and may change the treatment paradigm in years to come [76, 106].

**Table 1.** New Antifungals Recently Approved or in Advanced Stages of Clinical Development

Agent	Mechanism of Action	Spectrum	Considerations
Oteseconazole	Inhibition of fungal ergosterol synthase	*Candida* spp, dermatophytes, dimorphic fungi	Fewer adverse effects and drug-drug interactions. Extremely long half-life. Contraindicated in women of reproductive potential due to potential teratogenicity in some animal models.
Rezafungin	Inhibition of 1,3-β-d-glucan synthase	*Candida* spp, *Aspergillus* spp, *Pneumocystis jirovecii*	Longer half-life allowing weekly administration. Parenteral. Like other echinocandins, has excellent safety profile and no significant drug-drug interactions, poor penetration to central nervous system and urine.
Ibrexafungerp	Inhibition of 1,3-β-d-glucan synthase	*Candida* spp, *Aspergillus* spp, *Pneumocystis jirovecii*	Oral formulation, active against azole- and echinocandin-resistant *Candida* species
Olorofim	Inhibition of dihydroorotate dehydrogenase (involved in pyrimidine biosynthesis)	*Aspergillus* spp, *Lomentospora, Scedosporium* spp, other rare molds, *Coccidioides* spp, *Histoplasma* spp. No reliable activity against yeasts, Mucorales, *Fusarium solani*	Active against many drug-resistant molds that lack effective treatment options, good central nervous system penetration, limited drug-drug interactions anticipated. Hepatotoxicity in ∼10% of patients, requiring discontinuation in 2.5%.
Fosmanogepix	Inhibition of Gwt1 (involved in transport of mannoproteins)	*Candida* spp, *Aspergillus* spp, *Fusarium* spp, *Lomentospora/Scedosporium* spp *Cryptococcus* spp, *Coccidioides* spp	Novel mechanism of action; intravenous and oral; active against resistant fungi including *Candida* and *Aspergillus* species and rare molds such as *Lomentospora, Scedosporium*, and *Fusarium.* Good central nervous system penetration. Few drug-drug interactions anticipated.
Encochleated amphotericin B	Binds ergosterol, disrupting cell membranes	Broad-spectrum; same spectrum as Amphotericin B	Oral; improved bioavailability; decreased toxicities

Until now, treatment of invasive fungal infections has been limited to few classes of antifungals, namely the polyenes (amphotericin B), azoles, and echinocandins. Flucytosine is another antifungal with a limited role as part of combination therapy in some systemic and resistant mycoses (eg, in the central nervous system or urinary tract), and terbinafine is occasionally used in combination in difficult to treat mold infections. New agents among the azoles include oteseconazole, which specifically targets fungal ergosterol synthase, and is negligibly metabolized by mammalian CYP450 enzymes, resulting in a long half-life, fewer adverse effects, and avoidance of drug–drug interactions [107]. It has already received Food and Drug Administration approval for treatment of recurrent vulvovaginal candidiasis in women who are not of child-bearing potential [108]. Among the echinocandins, rezafungin has a longer half-life allowing once-weekly administration and has been approved for treatment of invasive candidiasis and candidemia [109]. Ibrexafungerp is an oral triterpenoid antifungal that shares a mechanism of action with echinocandins (inhibition of 1,3-β-d-glucan synthase) and that has been approved by the Food and Drug Administration for treatment of vulvovaginal candidiasis [108] and is currently being evaluated for other indications. Encochleated amphotericin B is a novel reformulation of the polyene within a lipid bilayer rolled into a taquito-shaped nanocochleate, which improves bioavailability that enables oral administration and an improved safety profile [110]. A third-generation renal-sparing polyene has also been developed and is undergoing preclinical evaluation [111].

In addition to these, there are several first-in-class antifungals with novel mechanisms of action. These include olorofim, an orotomide, which works by blocking pyrimidine biosynthesis and has potent antifungal activity against *Lomentospora, Scedosporium*, and azole-resistant *Aspergillus* isolates, and some dimorphic fungi [112]; and fosmanogepix, the first gepix antifungal, which inhibits glycosylphosphatidylinositol synthesis by binding to Gwt1, and which shows promise for a wide spectrum that includes yeasts (including *C auris*) but that is most anticipated for potent activity against *Lomentospora, Scedosporium, Fusarium*, and *Aspergillus* [104].

## LOOKING TO THE FUTURE: CHALLENGES FOR THE FIELD OF MYCOLOGY

### Expanding the Scope of Clinical Trials

The development of new treatments for fungal diseases has forced into focus issues of clinical trial design to identify the optimal roles of these agents in our armamentarium. The development of clinical trials for the treatment and prevention of fungal infections must consider input from a range of stakeholders, including patients, physicians, pathologists, pharmaceutical companies, and government agencies. Most clinical trials evaluating mold-active therapies are conducted in the global north and focus primarily on hematological oncology patients, which may limit generalizability of results beyond these populations. Trials for treatment of cryptococcal meningitis are generated in resource-limited settings in the context of HIV, although HIV-seronegative persons comprise most affected patients in resource-rich countries. In general, there is a need to explore management of IFDs in patients with other risk factors, including emerging risks from new antineoplastic therapeutics outlined previously. Moreover, most clinical trials are conducted only in nonpregnant adults and exclude children. It is imperative to improve global representation and equity in clinical trials for IFDs and to focus on groups in highest need.

### Clinical Trial Response Criteria: Opportunities for Improvement

Given the high associated mortality of IFD and its increased impact in populations of patients who are heavily immunosuppressed and often critically ill, the design and conduct of successful trials for novel antifungal therapeutics remains challenging [113–115]. In particular, the current response criteria for invasive fungal disease have important limitations that have spurred efforts to expand the definition of treatment success, better understand attributable mortality resulting from IFDs, incorporate mycologic biomarkers, and work toward the use of patient-reported outcomes.

### Where Clinical Trials Fall Short

Observational studies and the use of secondary data present new opportunities for mycology research because they can help answer questions that are difficult to address through randomized controlled trials. However, there are barriers to the acceptance and expertise needed to conduct adaptive trials in mycology. Cohort studies have gained traction in mycology, and the use of electronic data sources provides a wealth of data collected over many years, offering opportunities for exploring rare outcomes and exposures more rapidly than traditional methods. Although such data sources have been underused in mycology, they enable a wider range of research questions to be addressed in a timely manner. A tradeoff of using secondary data is that outcomes tend to be broad, but the benefits of this approach can be significant.

### Workforce Challenges

The challenges and opportunities in clinical mycology demand a robust workforce of clinicians and scientists focused on these problems. However, there is a common agreement that there are too few mycologists around the world, and few investigators enter and are retained in the field. Among medically trained clinical mycologists, most are infectious diseases (ID) physicians, and, to an extent, challenges in ensuring a robust ID workforce are reverberated in the clinical mycology arena. Challenges in attracting trainees to the subspecialty are well documented. For instance, one quarter of U.S. training posts for ID went unfilled in the residency match of 2023 [116]. Human Resources & Services Administration models suggest that there are at least 240 too few ID physicians in the United States, with shortfalls concentrated in rural areas [117]; in fact, an earlier study suggested that >200 million Americans live in counties with no or insufficient access to an ID physician [118].

## CONCLUSIONS

Invasive fungal disease represents a worldwide public health threat and key opportunities and challenges are facing the field of mycology over the coming years. Improved understanding of host risk factors, epidemiologic surveillance of emerging resistant fungal pathogens, and expansion of the clinical and basic research pipelines in mycology are essential to improve patient outcomes.

## Note

***Potential conflicts of interest*.** A. S. reports research funding from Astellas and Mayne and consulting fees from GSK and F2G. P. P. reports research support from Cidara, F2G, Scynexis, Astellas, Gilead, Merck; and consulting fees for Matinas, Cidara, Melinta, Basilea, TFF. T. M. reports research support from Cidara, T2 Biosystems, F2G, Scynexis; and consulting fees for Pfizer. All other authors report no potential conflicts.

## References

[1] World Health Organization . WHO fungal priority pathogens list to guide research, development and public health action. Geneva: World Health Organization, 2022. Licence: CC BY-NC-SA 3.0 IGO vol. 1.

[2] Neofytos D, Lu K, Hatfield-Seung A, et al Epidemiology, outcomes, and risk factors of invasive fungal infections in adult patients with acute myelogenous leukemia after induction chemotherapy. Diagn Microbiol Infect Dis 2013; 75:144–9. 23142166 10.1016/j.diagmicrobio.2012.10.001 PMC3986043

[3] Shoham S . Emerging fungal infections in solid organ transplant recipients. Infect Dis Clin North Am 2013; 27:305–16. 23714342 10.1016/j.idc.2013.02.004 PMC3690580

[4] Kontoyiannis DP, Marr KA, Park BJ, et al Prospective surveillance for invasive fungal infections in hematopoietic stem cell transplant recipients, 2001–2006: overview of the transplant-associated infection surveillance network (TRANSNET) database. Clin Infect Dis 2010; 50:1091–100. 20218877 10.1086/651263

[5] Baddley JW . Clinical risk factors for invasive aspergillosis. Med Mycol 2011; 49:S7–12. 20718606 10.3109/13693786.2010.505204

[6] Pagano L, Caira M, Nosari A, et al Fungal infections in recipients of hematopoietic stem cell transplants: results of the SEIFEM B-2004 study—Sorveglianza Epidemiologica Infezioni Fungine nelle Emopatie Maligne. Clin Infect Dis 2007; 45:1161–70. 17918077 10.1086/522189

[7] Herbrecht R, Bories P, Moulin JC, Ledoux MP, Letscher-Bru V. Risk stratification for invasive aspergillosis in immunocompromised patients. Ann N Y Acad Sci 2012; 1272:23–30. 23231711 10.1111/j.1749-6632.2012.06829.x

[8] Hoenigl M, Strenger V, Buzina W, et al European organization for the research and treatment of cancer/mycoses study group (EORTC/MSG) host factors and invasive fungal infections in patients with haematological malignancies. J Antimicrob Chemother 2012; 67:2029–33. 22566591 10.1093/jac/dks155

[9] Pappas PG, Lionakis MS, Arendrup MC, Ostrosky-Zeichner L, Kullberg BJ. Invasive candidiasis. Nat Rev Dis Primers 2018; 4:18026. 29749387 10.1038/nrdp.2018.26

[10] Morrison VA, Haake RJ, Weisdorf DJ. Non-Candida fungal infections after bone marrow transplantation: risk factors and outcome. Am J Med 1994; 96:497–503. 8017446 10.1016/0002-9343(94)90088-4

[11] Martin SI, Marty FM, Fiumara K, Treon SP, Gribben JG, Baden LR. Infectious complications associated with alemtuzumab use for lymphoproliferative disorders. Clin Infect Dis 2006; 43:16–24. 16758413 10.1086/504811

[12] Hammond SP, Marty FM, Bryar JM, DeAngelo DJ, Baden LR. Invasive fungal disease in patients treated for newly diagnosed acute leukemia. Am J Hematol 2010; 85:695–9. 20652970 10.1002/ajh.21776

[13] Nucci M, Marr KA. Emerging fungal diseases. Clin Infect Dis 2005; 41:521–6. 16028162 10.1086/432060

[14] Richardson M, Lass-Flörl C. Changing epidemiology of systemic fungal infections. Clin Microbiol Infect 2008; 14 Suppl 4:5–24. 10.1111/j.1469-0691.2008.01978.x 18430126

[15] Lewis RE, Cahyame-Zuniga L, Leventakos K, et al Epidemiology and sites of involvement of invasive fungal infections in patients with haematological malignancies: a 20-year autopsy study. Mycoses 2013; 56:638–45. 23551865 10.1111/myc.12081

[16] Lamoth F, Lewis RE, Walsh TJ, Kontoyiannis DP. Navigating the uncertainties of COVID-19-associated aspergillosis: a comparison with influenza-associated aspergillosis. J Infect Dis 2021; 224:1631–40. 33770176 10.1093/infdis/jiab163 PMC8083649

[17] Hsu AP, Korzeniowska A, Aguilar CC, et al Immunogenetics associated with severe coccidioidomycosis. JCI Insight 2022; 7: e159491. 36166305 10.1172/jci.insight.159491 PMC9746810

[18] Lionakis MS . Genetic susceptibility to fungal infections in humans. Curr Fungal Infect Rep 2012; 6:11–22. 23087779 10.1007/s12281-011-0076-4 PMC3475324

[19] Little JS, Weiss ZF, Hammond SP. Invasive fungal infections and targeted therapies in hematological malignancies. J Fungi (Basel) 2021; 7:1058. 34947040 10.3390/jof7121058 PMC8706272

[20] Davis JS, Ferreira D, Paige E, Gedye C, Boyle M. Infectious complications of biological and small molecule targeted immunomodulatory therapies. Clin Microbiol Rev 2020; 33:e00035-19. 32522746 10.1128/CMR.00035-19 PMC7289788

[21] Mikulska M, Lanini S, Gudiol C, et al ESCMID study Group for Infections in Compromised Hosts (ESGICH) Consensus Document on the safety of targeted and biological therapies: an infectious diseases perspective (Agents targeting lymphoid cells surface antigens [I]: CD19, CD20 and CD52). Clin Microbiol Infect 2018; 24 Suppl 2:S71–82. 29447988 10.1016/j.cmi.2018.02.003

[22] Reinwald M, Silva JT, Mueller NJ, et al ESCMID study Group for Infections in Compromised Hosts (ESGICH) Consensus Document on the safety of targeted and biological therapies: an infectious diseases perspective (intracellular signaling pathways: tyrosine kinase and mTOR inhibitors). Clin Microbiol Infect 2018; 24 Suppl 2:S53–70. 29454849 10.1016/j.cmi.2018.02.009

[23] Aguilar-Company J, Fernández-Ruiz M, García-Campelo R, Garrido-Castro AC, Ruiz-Camps I. ESCMID study Group for Infections in Compromised Hosts (ESGICH) Consensus Document on the safety of targeted and biological therapies: an infectious diseases perspective (cell surface receptors and associated signaling pathways). Clin Microbiol Infect 2018; 24 Suppl 2:S41–52. 29426804 10.1016/j.cmi.2017.12.027

[24] Chamilos G, Lionakis MS, Kontoyiannis DP. Call for action: invasive fungal infections associated with ibrutinib and other small molecule kinase inhibitors targeting immune signaling pathways. Clin Infect Dis 2018; 66:140–8. 29029010 10.1093/cid/cix687 PMC5850040

[25] Jagasia M, Perales M-A, Schroeder MA, et al Ruxolitinib for the treatment of steroid-refractory acute GVHD (REACH1): a multicenter, open-label phase 2 trial. Blood 2018; 135:1739–49. 10.1182/blood.2020004823 PMC7229262 32160294

[26] Xuan L, Wang Y, Huang F, et al Sorafenib maintenance in patients with FLT3-ITD acute myeloid leukaemia undergoing allogeneic haematopoietic stem-cell transplantation: an open-label, multicentre, randomised phase 3 trial. Lancet Oncol 2020; 21:1201–12. 32791048 10.1016/S1470-2045(20)30455-1

[27] Garcia JS, Kim HT, Brock J, et al Maintenance therapy with venetoclax/azacitidine can be safely given after venetoclax/FluBu2 RIC allogeneic transplantation for the treatment of high risk MDS/AML: results of a phase 1 study. Blood 2022; 140:917–9.

[28] Vanneman M, Dranoff G. Combining immunotherapy and targeted therapies in cancer treatment. Nat Rev Cancer 2012; 12:237–51. 22437869 10.1038/nrc3237 PMC3967236

[29] Watkins B, Qayed M, McCracken C, et al Phase II trial of costimulation blockade with Abatacept for prevention of acute GVHD. J Clin Oncol 2021; 39:1865–77. 33449816 10.1200/JCO.20.01086 PMC8260909

[30] Sharman JP, Egyed M, Jurczak W, et al Acalabrutinib with or without obinutuzumab versus chlorambucil and obinutuzmab for treatment-naive chronic lymphocytic leukaemia (ELEVATE TN): a randomised, controlled, phase 3 trial. Lancet 2020; 395:1278–91. 32305093 10.1016/S0140-6736(20)30262-2 PMC8151619

[31] Jones JA, Robak T, Brown JR, et al Efficacy and safety of idelalisib in combination with ofatumumab for previously treated chronic lymphocytic leukaemia: an open-label, randomised phase 3 trial. Lancet Haematol 2017; 4:e114–26. 28257752 10.1016/S2352-3026(17)30019-4

[32] Zelenetz AD, Barrientos JC, Brown JR, et al Idelalisib or placebo in combination with bendamustine and rituximab in patients with relapsed or refractory chronic lymphocytic leukaemia: interim results from a phase 3, randomised, double-blind, placebo-controlled trial. Lancet Oncol 2017; 18:297–311. 28139405 10.1016/S1470-2045(16)30671-4 PMC5589180

[33] Rajewsky K . The advent and rise of monoclonal antibodies. Nature 2019; 575:47–9. 31686050 10.1038/d41586-019-02840-w

[34] Mullard A . FDA approves 100th monoclonal antibody product. Nat Rev Drug Discov 2021; 20:491–5. 33953368 10.1038/d41573-021-00079-7

[35] Redelman-Sidi G, Michielin O, Cervera C, et al ESCMID study Group for Infections in Compromised Hosts (ESGICH) Consensus Document on the safety of targeted and biological therapies: an infectious diseases perspective (immune checkpoint inhibitors, cell adhesion inhibitors, sphingosine-1-phosphate receptor modulators and proteasome inhibitors). Clin Microbiol Infect 2018; 24(Suppl 2):S95–107. 29427804 10.1016/j.cmi.2018.01.030 PMC5971148

[36] Abers MS, Lionakis MS. Infectious complications of immune checkpoint inhibitors. Infect Dis Clin North Am 2020; 34:235–43. 32334989 10.1016/j.idc.2020.02.004

[37] Vallabhaneni S, Chiller TM. Fungal infections and new biologic therapies. Curr Rheumatol Rep 2016; 18:29. 27032792 10.1007/s11926-016-0572-1

[38] Bongartz T, Sutton AJ, Sweeting MJ, Buchan I, Matteson EL, Montori V. Anti-TNF antibody therapy in rheumatoid arthritis and the risk of serious infections and malignancies: systematic review and meta-analysis of rare harmful effects in randomized controlled trials. JAMA 2006; 295:2275–85. 16705109 10.1001/jama.295.19.2275

[39] Filler SG, Yeaman MR, Sheppard DC. Tumor necrosis factor inhibition and invasive fungal infections. Clin Infect Dis 2005; 41 Suppl 3:S208–12. 15983902 10.1086/430000

[40] Bergstrom L, Yocum DE, Ampel NM, et al Increased risk of coccidioidomycosis in patients treated with tumor necrosis factor α antagonists. Arthritis Rheum 2004; 50:1959–66. 15188373 10.1002/art.20454

[41] Lee JH, Slifman NR, Gershon SK, et al Life-threatening histoplasmosis complicating immunotherapy with tumor necrosis factor α antagonists infliximab and etanercept. Arthritis Rheum 2002; 46:2565–70. 12384912 10.1002/art.10583

[42] Novosad SA, Winthrop KL. Beyond tumor necrosis factor inhibition: the expanding pipeline of biologic therapies for inflammatory diseases and their associated infectious sequelae. Clin Infect Dis 2014; 58:1587–98. 24585557 10.1093/cid/ciu104

[43] Vergidis P, Avery RK, Wheat LJ, et al Histoplasmosis complicating tumor necrosis factor-α blocker therapy: a retrospective analysis of 98 cases. Clin Infect Dis 2015; 61:409–17. 25870331 10.1093/cid/civ299 PMC4796723

[44] Smith JA, Kauffman CA. Endemic fungal infections in patients receiving tumour necrosis factor-α inhibitor therapy. Drugs 2009; 69:1403–15. 19634920 10.2165/00003495-200969110-00002

[45] Winthrop KL, Abrams M, Yakrus M, et al An outbreak of mycobacterial furunculosis associated with footbaths at a nail salon. N Engl J Med 2002; 346:1366–71. 11986410 10.1056/NEJMoa012643

[46] Maertens J, Cesaro S, Maschmeyer G, et al ECIL guidelines for preventing Pneumocystis jirovecii pneumonia in patients with haematological malignancies and stem cell transplant recipients. J Antimicrob Chemother 2016; 71:2397–404. 27550992 10.1093/jac/dkw157

[47] Okada S, Puel A, Casanova J-L, Kobayashi M. Chronic mucocutaneous candidiasis disease associated with inborn errors of IL-17 immunity. Clin Transl Immunology 2016; 5:e114. 28090315 10.1038/cti.2016.71 PMC5192062

[48] Break TJ, Oikonomou V, Dutzan N, et al Aberrant type 1 immunity drives susceptibility to mucosal fungal infections. Science 2021; 371:eaay5731. 33446526 10.1126/science.aay5731 PMC8326743

[49] Winthrop KL, Mariette X, Silva JT, et al ESCMID study Group for Infections in Compromised Hosts (ESGICH) Consensus Document on the safety of targeted and biological therapies: an infectious diseases perspective (soluble immune effector molecules [II]: agents targeting interleukins, immunoglobulins and complement factors). Clin Microbiol Infect 2018; 24:S21–40. 29447987 10.1016/j.cmi.2018.02.002

[50] Bedard PL, Hyman DM, Davids MS, Siu LL. Small molecules, big impact: 20 years of targeted therapy in oncology. Lancet 2020; 395:1078–88. 32222192 10.1016/S0140-6736(20)30164-1

[51] Fiorcari S, Maffei R, Vallerini D, et al BTK inhibition impairs the innate response against fungal infection in patients with chronic lymphocytic leukemia. Front Immunol 2020; 11:2158. 32983178 10.3389/fimmu.2020.02158 PMC7485008

[52] Chan TSY, Au-Yeung R, Chim CS, Wong SCY, Kwong YL. Disseminated fusarium infection after ibrutinib therapy in chronic lymphocytic leukaemia. Ann Hematol 2017; 96:871–2. 28184982 10.1007/s00277-017-2944-7

[53] Arthurs B, Wunderle K, Hsu M, Kim S. Invasive aspergillosis related to ibrutinib therapy for chronic lymphocytic leukemia. Respir Med Case Rep 2017; 21:27–9. 28377877 10.1016/j.rmcr.2017.03.011 PMC5369366

[54] Anastasopoulou A, DiPippo AJ, Kontoyiannis DP. Non-Aspergillus invasive mould infections in patients treated with ibrutinib. Mycoses 2020; 63:787–93. 32458510 10.1111/myc.13120

[55] Varughese T, Taur Y, Cohen N, et al Serious infections in patients receiving ibrutinib for treatment of lymphoid cancer. Clin Infect Dis 2018; 67:687–92. 29509845 10.1093/cid/ciy175 PMC6093991

[56] Lionakis MS, Dunleavy K, Roschewski M, et al Inhibition of B cell receptor signaling by ibrutinib in primary CNS lymphoma. Cancer Cell 2017; 31:833–843.e5. 28552327 10.1016/j.ccell.2017.04.012 PMC5571650

[57] Cummins KC, Cheng MP, Kubiak DW, Davids MS, Marty FM, Issa NC. Isavuconazole for the treatment of invasive fungal disease in patients receiving ibrutinib. Leuk Lymphoma 2019; 60:527–30. 30037292 10.1080/10428194.2018.1485913

[58] Rogers KA, Mousa L, Zhao Q, et al Incidence of opportunistic infections during ibrutinib treatment for B-cell malignancies. Leukemia 2019; 33:2527–30. 31086260 10.1038/s41375-019-0481-1 PMC7425823

[59] Rogers KA, Luay M, Zhao Q, et al Incidence and type of opportunistic infections during ibrutinib treatment at a single academic center. Blood 2017; 130:830.

[60] Ghez D, Calleja A, Protin C, et al Early-onset invasive aspergillosis and other fungal infections in patients treated with ibrutinib. Blood 2018; 131:1955–9. 29437588 10.1182/blood-2017-11-818286

[61] Tillman BF, Pauff JM, Satyanarayana G, Talbott M, Warner JL. Systematic review of infectious events with the Bruton tyrosine kinase inhibitor ibrutinib in the treatment of hematologic malignancies. Eur J Haematol 2018; 100:325–34. 29285806 10.1111/ejh.13020

[62] Maschmeyer G, De Greef J, Mellinghoff SC, et al Infections associated with immunotherapeutic and molecular targeted agents in hematology and oncology. A position paper by the European Conference on Infections in Leukemia (ECIL). Leukemia 2019; 33:844–62. 30700842 10.1038/s41375-019-0388-x PMC6484704

[63] Cuneo A, Barosi G, Danesi R, et al Management of adverse events associated with idelalisib treatment in chronic lymphocytic leukemia and follicular lymphoma: a multidisciplinary position paper. Hematol Oncol 2019; 37:3–14. 30187496 10.1002/hon.2540 PMC6585802

[64] Cheah CY, Fowler NH. Idelalisib in the management of lymphoma. Blood 2016; 128:331–6. 27252232 10.1182/blood-2016-02-702761 PMC5161010

[65] Bird ST, Tian F, Flowers N, et al Idelalisib for treatment of relapsed follicular lymphoma and chronic lymphocytic leukemia: a comparison of treatment outcomes in clinical trial participants vs medicare beneficiaries. JAMA Oncol 2020; 6:248–54. 31855259 10.1001/jamaoncol.2019.3994 PMC6990831

[66] Oncologic Drugs Advisory Committee Meeting. Phosphatidylinositol 3-Kinase (PI3K) Inhibitors in Hematologic Malignancies. Food and Drug Administration. 2022. Available at: https://www.fda.gov/media/159332/download. Accessed November 1, 2023.

[67] Little JS, Aleissa MM, Beluch K, et al Low incidence of invasive fungal disease following CD19 chimeric antigen receptor T-cell therapy for non-Hodgkin lymphoma. Blood Adv 2022; 6:4821–30. 35802461 10.1182/bloodadvances.2022007474 PMC9631654

[68] Kampouri E, Little JS, Rejeski K, Manuel O, Hammond SP, Hill JA. Infections after chimeric antigen receptor (CAR)-T-cell therapy for hematologic malignancies. Transpl Infect Dis 2023; 25 Suppl 1:e14157. 37787373 10.1111/tid.14157

[69] Hill JA, Seo SK. How I prevent infections in patients receiving CD19-targeted chimeric antigen receptor T cells for B-cell malignancies. Blood 2020; 136:925–35. 32582924 10.1182/blood.2019004000 PMC7441168

[70] Garner W, Samanta P, Haidar G. Invasive fungal infections after anti-cd19 chimeric antigen receptor-modified T-cell therapy: state of the evidence and future directions. J Fungi (Basel) 2021; 7:1–15. 10.3390/jof7020156 PMC7927024 33672208

[71] Haidar G, Garner W, Hill JA. Infections after anti-CD19 chimeric antigen receptor T-cell therapy for hematologic malignancies: timeline, prevention, and uncertainties. Curr Opin Infect Dis 2020; 33:449–57. 33009139 10.1097/QCO.0000000000000679

[72] Locke FL, Miklos DB, Jacobson CA, et al Axicabtagene ciloleucel as second-line therapy for large B-cell lymphoma. N Engl J Med 2022; 386:640–54. 34891224 10.1056/NEJMoa2116133

[73] Mougiakakos D, Krönke G, Völkl S, et al CD19-targeted CAR T cells in refractory systemic lupus erythematosus. N Engl J Med 2021; 385:567–9. 34347960 10.1056/NEJMc2107725

[74] Maus MV, Lionakis MS. Infections associated with the new ‘nibs and mabs’ and cellular therapies. Curr Opin Infect Dis 2020; 33:281–9. 32657964 10.1097/QCO.0000000000000656 PMC7367497

[75] Lionakis MS, Iliev ID, Hohl TM. Immunity against fungi. JCI Insight 2017; 2:e93156. 28570272 10.1172/jci.insight.93156 PMC5453709

[76] Lionakis MS, Drummond RA, Hohl TM. Immune responses to human fungal pathogens and therapeutic prospects. Nat Rev Immunol 2023; 23:433–52. 36600071 10.1038/s41577-022-00826-w PMC9812358

[77] Hsu AP, Sampaio EP, Khan J, et al Mutations in GATA2 are associated with the autosomal dominant and sporadic monocytopenia and mycobacterial infection (MonoMAC) syndrome. Blood 2011; 118:2653–5. 21670465 10.1182/blood-2011-05-356352 PMC3172785

[78] Vinh DC, Patel SY, Uzel G, et al Autosomal dominant and sporadic monocytopenia with susceptibility to mycobacteria, fungi, papillomaviruses, and myelodysplasia. Blood 2010; 115:1519–29. 20040766 10.1182/blood-2009-03-208629 PMC2830758

[79] Rosenzweig SD, Holland SM. Defects in the interferon-γ and interleukin-12 pathways. Immunol Rev 2005; 203:38–47. 15661020 10.1111/j.0105-2896.2005.00227.x

[80] Bochud P-Y, Chien JW, Marr KA, et al Toll-like receptor 4 polymorphisms and aspergillosis in stem-cell transplantation. N Engl J Med 2008; 359:1766–77. 18946062 10.1056/NEJMoa0802629 PMC2656610

[81] Schauwvlieghe AFAD, Rijnders BJA, Philips N, et al Invasive aspergillosis in patients admitted to the intensive care unit with severe influenza: a retrospective cohort study. Lancet Respir Med 2018; 6:782–92. 30076119 10.1016/S2213-2600(18)30274-1

[82] Baddley JW, Thompson GR, Chen SC-A, et al Coronavirus disease 2019-associated invasive fungal infection. Open Forum Infect Dis 2021; 8:ofab510. 34877364 10.1093/ofid/ofab510 PMC8643686

[83] Hoenigl M, Seidel D, Sprute R, et al COVID-19-associated fungal infections. Nat Microbiol 2022; 7:1127–40. 35918423 10.1038/s41564-022-01172-2 PMC9362108

[84] Kariyawasam RM, Dingle TC, Kula BE, Vandermeer B, Sligl WI, Schwartz IS. Defining COVID-19–associated pulmonary aspergillosis: systematic review and meta-analysis. Clin Microbiol Infect 2022; 28:920–7. 35150878 10.1016/j.cmi.2022.01.027 PMC8828380

[85] Stone N, Gupta N, Schwartz I. Mucormycosis: time to address this deadly fungal infection. Lancet Microbe 2021; 2:e343–4. 35544192 10.1016/S2666-5247(21)00148-8

[86] Sen M, Honavar SG, Bansal R, et al Epidemiology, clinical profile, management, and outcome of COVID-19-associated rhino-orbital-cerebral mucormycosis in 2826 patients in India—Collaborative OPAI-IJO Study on Mucormycosis in COVID-19 (COSMIC), report 1. Indian J Ophthalmol 2021; 69:1670–92. 34156034 10.4103/ijo.IJO_1565_21 PMC8374756

[87] Smith RM, Schaefer MK, Kainer MA, et al Fungal infections associated with contaminated methylprednisolone injections. N Engl J Med 2013; 369:1598–609. 23252499 10.1056/NEJMoa1213978

[88] Strong N, Meeks G, Sheth SA, et al Neurovascular complications of iatrogenic fusarium solani meningitis. N Engl J Med 2024; 390:522–9. 38324485 10.1056/NEJMoa2308192 PMC11905984

[89] García-Rodríguez G, Duque-Molina C, Kondo-Padilla I, et al Outbreak of Fusarium solani meningitis in immunocompetent persons associated with neuraxial blockade in Durango, Mexico, 2022–2023. Open Forum Infect Dis 2024; 11:ofad690. 38370296 10.1093/ofid/ofad690 PMC10873708

[90] Friedman DZP, Schwartz IS. Emerging fungal infections: new patients, new patterns, and new pathogens. J Fungi (Basel) 2019; 5:67. 31330862 10.3390/jof5030067 PMC6787706

[91] Havlickova B, Czaika VA, Friedrich M. Epidemiological trends in skin mycoses worldwide. Mycoses 2008; 51 Suppl 4:2–15. 18783559 10.1111/j.1439-0507.2008.01606.x

[92] Gu D, Hatch M, Ghannoum M, Elewski BE. Treatment-resistant dermatophytosis: a representative case highlighting an emerging public health threat. JAAD Case Rep 2020; 6:1153–5. 33134459 10.1016/j.jdcr.2020.05.025 PMC7591325

[93] Cañete-Gibas CF, Mele J, Patterson HP, et al Terbinafine-resistant dermatophytes and the presence of trichophyton indotineae in North America. J Clin Microbiol 2023; 61:e0056223. 37432126 10.1128/jcm.00562-23 PMC10446870

[94] Gupta AK, Venkataraman M, Hall DC, Cooper EA, Summerbell RC. The emergence of Trichophyton indotineae: implications for clinical practice. Int J Dermatol 2023; 62:857–61. 35867962 10.1111/ijd.16362

[95] Wiederhold NP, Verweij PE. Aspergillus fumigatus and pan-azole resistance: who should be concerned? Curr Opin Infect Dis 2020; 33:290–7. 32657965 10.1097/QCO.0000000000000662

[96] Lestrade PPA, Buil JB, van der Beek MT, et al Paradoxical trends in azole-resistant aspergillus fumigatus in a national multicenter surveillance program, The Netherlands, 2013–2018. Emerg Infect Dis 2020; 26:1447–55. 32568033 10.3201/eid2607.200088 PMC7323544

[97] Pham CD, Reiss E, Hagen F, Meis JF, Lockhart SR. Passive surveillance for azole-resistant aspergillus fumigatus, United States, 2011–2013. Emerg Infect Dis 2014; 20:1498. 25148217 10.3201/eid2009.140142 PMC4178384

[98] Lestrade PP, Bentvelsen RG, Schauwvlieghe AFAD, et al Voriconazole resistance and mortality in invasive aspergillosis: a multicenter retrospective cohort study. Clin Infect Dis 2019; 68:1463–71. 30307492 10.1093/cid/ciy859

[99] Lass-Flörl C, Dietl A-M, Kontoyiannis DP, Brock M. Aspergillus terreus species complex. Clin Microbiol Rev 2021; 34:e0031120. 34190571 10.1128/CMR.00311-20 PMC8404697

[100] Lyman M, Forsberg K, Sexton DJ, et al Worsening spread of Candida auris in the United States, 2019 to 2021. Ann Intern Med 2023; 176:489–95. 36940442 10.7326/M22-3469 PMC11307313

[101] Simon SP, Li R, Silver M, et al Comparative outcomes of Candida auris bloodstream infections: a multicenter retrospective case-control study. Clin Infect Dis 2023; 76:E1436–43. 36062367 10.1093/cid/ciac735

[102] Forsberg K, Woodworth K, Walters M, et al Candida auris: the recent emergence of a multidrug-resistant fungal pathogen. Med Mycol 2019; 57:e7. 30085270 10.1093/mmy/myy054

[103] Rauseo AM, Coler-Reilly A, Larson L, Spec A. Hope on the horizon: novel fungal treatments in development. Open Forum Infect Dis 2020; 7:ofaa016. 32099843 10.1093/ofid/ofaa016 PMC7031074

[104] Gintjee TJ, Donnelley MA, Thompson GR III. Aspiring antifungals: review of current antifungal pipeline developments. J Fungi (Basel) 2020; 6:28. 32106450 10.3390/jof6010028 PMC7151215

[105] Friedman DZP, Schwartz IS. Emerging diagnostics and therapeutics for invasive fungal infections. Infect Dis Clin North Am 2023; 37:593–616. 37532392 10.1016/j.idc.2023.05.001

[106] Wurster S, Watowich SS, Kontoyiannis DP. Checkpoint inhibitors as immunotherapy for fungal infections: promises, challenges, and unanswered questions. Front Immunol 2022; 13: 1018202. 36389687 10.3389/fimmu.2022.1018202 PMC9640966

[107] Martens MG, Maximos B, Degenhardt T, et al Phase 3 study evaluating the safety and efficacy of oteseconazole in the treatment of recurrent vulvovaginal candidiasis and acute vulvovaginal candidiasis infections. Am J Obstet Gynecol 2022; 227:880.e1–e11. 10.1016/j.ajog.2022.07.023 35863457

[108] Sobel JD . New antifungals for vulvovaginal candidiasis: what is their role? Clin Infect Dis 2023; 76:783–5. 36610791 10.1093/cid/ciad002

[109] Thompson GR III, Soriano A, Honore PM, et al Efficacy and safety of rezafungin and caspofungin in candidaemia and invasive candidiasis: pooled data from two prospective randomised controlled trials. Lancet Infect Dis 2023; 24:319–28. 38008099 10.1016/S1473-3099(23)00551-0

[110] Boulware DR, Atukunda M, Kagimu E, et al Oral lipid nanocrystal amphotericin B for cryptococcal meningitis: a randomized clinical trial. Clin Infect Dis 2023; 77:1659–67. 37606364 10.1093/cid/ciad440 PMC10724459

[111] Maji A, Soutar CP, Zhang J, et al Tuning sterol extraction kinetics yields a renal-sparing polyene antifungal. Nature 2023; 623:1079–85. 37938782 10.1038/s41586-023-06710-4 PMC10883201

[112] Wiederhold NP . Review of the novel investigational antifungal olorofim. J Fungi (Basel) 2020; 6:1–11. 10.3390/jof6030122 PMC7557671 32751765

[113] Segal BH, Herbrecht R, Stevens DA, et al Defining responses to therapy and study outcomes in clinical trials of invasive fungal diseases: mycoses Study Group and European Organization for Research and Treatment of Cancer Consensus Criteria. Clin Infect Dis 2008; 47:674–83. 18637757 10.1086/590566 PMC2671230

[114] Slavin MA, Chen Y-C, Cordonnier C, et al When to change treatment of acute invasive aspergillosis: an expert viewpoint. J Antimicrob Chemother 2022; 77:16–23. 10.1093/jac/dkab317 PMC8730679 34508633

[115] Colombo AL, De Almeida JN, Lewis RE, Kontoyiannis DP. Quandaries of deciding when to change first-line therapy in invasive pulmonary aspergillosis. J Antimicrob Chemother 2022; 77:2897–900. 36059133 10.1093/jac/dkac301

[116] Feller Stephen . Nearly half of ID fellowship programs go unfilled as shortage persists. Available at: https://www.healio.com/news/infectious-disease/20231130/nearly-half-of-id-fellowship-programs-go-unfilled-as-shortage-persists. Accessed 1 December 2023.

[117] Health Resources and Services Administration, U. S. Department of Health and Human Services. Workforce Projections. data.hrsa.gov Available at: https://data.hrsa.gov/topics/health-workforce/workforce-projections. 2022.

[118] Walensky RP, McQuillen DP, Shahbazi S, Goodson JD. Where is the ID in COVID-19? Ann Intern Med 2020; 173:587–9. 32491920 10.7326/M20-2684 PMC7277486

